# Paradigms and Controversies in the Treatment of HIV-Related Burkitt Lymphoma

**DOI:** 10.1155/2012/403648

**Published:** 2012-04-18

**Authors:** Adam M. Petrich, Joseph A. Sparano, Samir Parekh

**Affiliations:** ^1^Division of Hematology/Oncology, Northwestern University, Chicago, IL 60611, USA; ^2^Department of Oncology, Montefiore Medical Center, Bronx, NY 10461, USA; ^3^Alert Einstein Cancer Center, Jack and Pearl Resnick Campus, 1300 Morris Park Avenue, Room 302, Bronx, NY 10461, USA

## Abstract

Burkitt lymphoma (BL) is a very aggressive subtype of non-Hodgkin's lymphoma that occurs with higher frequency in patients with HIV/AIDS. Patients with HIV-related BL (HIV-BL) are usually treated with high-intensity, multi-agent chemotherapy regimens. The addition of the monoclonal antibody Rituximab to chemotherapy has also been studied in this setting. The potential risks and benefits of commonly used regimens are reviewed herein, along with a discussion of controversial issues in the practical management of HIV-BL, including concurrent anti-retroviral therapy, treatment of relapsed and/or refractory disease, and the role of stem cell transplantation.

## 1. Introduction

Burkitt lymphoma (BL) is a very aggressive subtype of non-Hodgkin lymphoma (NHL) usually associated with translocation of the MYC oncogene. The World Health Organization (WHO) classification recognizes three clinical variants of BL: sporadic, endemic, and immunodeficiency related [1]. The last of these is particularly common in patients with human immunodeficiency virus (HIV), in whom the lifetime incidence of BL has been estimated at 10–20% [2], and wherein it constitutes an acquired immunodeficiency-syndrome- (AIDS-) defining illness.

The difference in clinical variants of BL may be explained by variation in stage of B-cell development at which lymphomagenesis occurs and by a potential relationship with Epstein Barr virus (EBV). It has been shown, for instance, that cases of endemic and AIDS-related BL (both of which are generally EBV related) have considerably highermutation rates than those of sporadic BL; EBV-positive BLs also have higher levels of somatic hypermutation of their variable region heavy chain genes, and evidence of antigen selection (whereas EBV-negative BLs generally fail to show this selection)[3]. These data suggest that EBV-negative BL arises from an early centroblast, while EBV-positive BL arises later in development, likely from a memory B cell or late germinal center B cell. Gene expression signatures of the three variants also appear to be distinct, with differences between endemic and sporadic cases of BL in terms of expression of proteins that influence the oncogenic potential of MYC [4], ectopic expression of which is a near-universal phenomenon in BL.

Historically, HIV/AIDS-related BL (HIV-BL) has represented a therapeutic challenge, mainly due to (a) the toxicity involved in treating HIV-positive patients with very intense and immunosuppressive regimens found to be successful in HIV-negative patients with BL, (b) the paucity of data from randomized controlled trials, and (c) the relatively small number of patients with this disease. As we will explore in this paper, several paradigms exist with respect to effective treatment of HIV-BL, though controversies and questions remain. The goal of this paper is to review the data behind several chemotherapeutic regimens and discuss several issues of particular relevance to this population.

## 2. Chemotherapy Regimens

The evolution of therapy for BL (reviewed by Magrath [5]) dates to at least 20 years before the first cases of HIV were reported, and the multiagent regimens devised during that time have formed the basis for trials in HIV-BL. In the 1970s, it was shown that treatment with short-course, high-intensity, multiagent chemotherapy, built on a backbone of cyclophosphamide, vincristine, and methotrexate, could result in substantial rates of durable remission [6] and that repeated intrathecal (IT) chemotherapy seemed important to the prevention of central nervous system (CNS) relapse [7]. When similar approaches were taken with HIV-positive patients [8, 9], similarly high rates of complete response (CR) were achieved. A trend toward inferior 5-year overall survival (OS) was appreciated in the HIV-positive patients, and was attributed to delayed complications of HIV, such as Kaposi's sarcoma and opportunistic infections. Importantly, treatment-related toxicity and mortality did not seem to be appreciably increased in the HIV-positive population. These data helped pave the way for further investigation of intensive therapy for patients with HIV-BL.

### 2.1. Hyper-CVAD

In 2002, Cortes et al. [10] reported their experience using Hyper-CVAD (hyperfractionated cyclophosphamide, vincristine, doxorubicin, and dexamethasone, in cycles alternating with methotrexate and cytarabine), along with highly active antiretroviral therapy (HAART) in patients with AIDS-related Burkitt lymphoma (*n* = 6) and leukemia (*n* = 7). Ten patients were over the age of 35, and nine were diagnosed with HIV at time of diagnosis of BL; median CD4+ T-cell (CD4) count was 77/*μ*L. Patients received a median of 6 cycles of therapy, though over 20% of cycles were delayed or reduced due to toxicity, and 35% were complicated by fever or infection. Interestingly, of seven patients who were on HAART or started during the first course, six remained alive in CR at time of publication, whereas all four patients who did not receive HAART died (three of causes related to progression of HIV-BL).

 The data for the addition of rituximab to this regimen (R-Hyper-CVAD) for HIV-BL remains somewhat lacking. The same group reported improved outcomes using R-Hyper-CVAD, as compared to historical cohorts treated with Hyper-CVAD without rituximab [11]. However, 45% of the patients included were diagnosed with B-cell acute lymphoblastic leukemia, as opposed to typical BL. When reporting on six HIV-BL patients included in the study, in abstract form at the 2003 meeting of the American Society of Clinical Oncology (ASCO) [12], they noted three deaths in CR due to HIV-related malignancy or infection.

### 2.2. CODOX-M/IVAC

Using the CODOX-M/IVAC regimen (dose-intense cyclophosphamide, doxorubicin, high-dose methotrexate/ifosfamide, etoposide, and high-dose cytarabine) in HIV-negative adults (*n* = 20) with BL, Magrath and colleagues reported 100% 2-year EFS [13], but at the cost of often severe neurotoxicity. In 2003, Wang and colleagues [14] reported their single-institution experience with CODOX-M/IVAC for patients with HIV-BL. When analyzing HIV-BL patients treated with CODOX-M/IVAC (*n* = 8) to those treated with less intense regimens (*n* = 6), they noted similar rates of CR (63% versus 67%, resp.) and 2-year EFS (57% versus 60%, resp.) in spite of disproportionately more high-risk patients in the CODOX-M/IVAC cohort (88% versus 33%). Furthermore, when all HIV-BL patients (*n* = 14) were analyzed (irrespective of treatment regimen) in comparison to HIV-negative patients with BL treated in similar fashion over the same time period (*n* = 24), HIV status affected rates of neither CR (HIV-positive patients, 64%; HIV-negative patients, 63%) nor 2-year EFS (HIV-positive patients, 59%; HIV-negative patients, 62%). The authors concluded that CODOX-M/IVAC (a) may overcome high-risk features in HIV-BL, and (b) did not appear significantly more toxic in patients with HIV-BL as compared to HIV-negative patients with BL.

CODOX-M/IVAC was subsequently modified by Lacasce and colleagues [15] by decreasing the dose of methotrexate, capping the vincristine dose, modifying the dose schedule of cyclophosphamide (to permit for earlier use of growth factor), and increasing the doxorubicin dose, all for the sake of preserving efficacy while reducing risk of neurotoxicity. In so doing, the authors realized a 2-year OS of 71% in HIV-negative patients with BL (*n* = 14), while observing seemingly significantly lower rates of neurotoxicity. This so-called “modified Magrath regimen,” constitutes with slight additional modification (including moving high-dose methotrexate from day 10 to day 15 in order to minimize toxicity), constitutes the chemotherapeutic backbone of an ongoing AIDS Malignancy Consortium (AMC) trial (AMC 048), which also adds rituximab to all cycles of therapy. At a median follow-up of nine months, with 34 patients treated, the authors reported no treatment-related mortality (TRM) and one-year OS of 82% [16].

### 2.3. Dose-Adjusted EPOCH

In spite of the beneficial effects of HAART upon incidence and outcome of ARL, concerns remained over the interaction between cytotoxic chemotherapy and agents included in HAART [17]. With this in mind, the National Institutes of Health (NIH) explored the use of infusional dose-adjusted etoposide, prednisone, vincristine, cyclophosphamide, and doxorubicin (DA-EPOCH) in ARL, with suspension of HAART for the duration of chemotherapy (planned 6 cycles) [18]. DA-EPOCH was chosen because (a) pre-clinical data suggested that sustained concentrations of doxorubicin, vincristine, and etoposide resulted in relatively less tumor resistance [19], and (b) inter-cycle dose adjustment permitted for maintenance of dose intensity while ameliorating toxicity [18]. In this trial, seven of 39 patients (18%) had BL, and those with BL had a significantly higher rate of CNS involvement versus those with diffuse large B-cell lymphoma (DLBCL; 4/7 versus 5/31, resp., *P* = 0.04). Although reporting of the BL subset was otherwise lacking, the trend toward inferior 53-month survival observed for BL, as compared to DLBCL (43% versus 66%, *P* = 0.22), was attributed to the high rate of CNS disease, as each patient with BL who died during follow-up was known to have CNS involvement prior to therapy.

 Success with EPOCH led NIH investigators to evaluate the addition of rituximab to this regimen in HIV-BL. It had been observed that high levels of response and OS could be maintained by giving short-course EPOCH with dose-dense rituximab (SC-EPOCH-RR), with number of courses determined by interim positron emission tomography-computed axial tomography (PET-CT) staging, to patients with HIV-related DLBCL [20]. This so-called “short course” consisted of three cycles of EPOCH-RR, followed by PET-CT, after which those with negative results received one additional cycle, and those with evidence of persistent disease received an additional 2-3 cycles. In HIV-DLBCL, OS of 73% at 53 months of follow-up was achieved [20]. Based on these results, the authors turned their attention to PET-CT-directed DA-EPOCH-R in HIV-BL and reported results as recently as 2009 [21], wherein 8 patients had been treated with 100% CR rate and OS and no TRM. Results updated as of September 29, 2011, indicate that each patient was treated with either three or four total cycles of DA-EPOCH-RR, and all remain in CR [22]. Of note no patients in this trial had CNS involvement, and suspension of HAART was a requirement.

 The AMC evaluated the addition of rituximab to DA-EPOCH for patients with newly diagnosed ARL in a randomized phase II trial (AMC 034 [23]), in which patients received either rituximab concurrently with each cycle (Arm A) or weekly for six cycles upon completion of DA-EPOCH (Arm B). DA-EPOCH was administered for two cycles beyond CR for a total of between 4 and 6 cycles. Although most patients had HIV-DLBCL, a significant proportion (27/106; 25%) had HIV-BL. When the response rate was analyzed in all treated patients who had DLBCL, CR occurred in 25 of 35 patients (71%; 95% CI, 54%–85%) in the concurrent arm and 20 of 44 patients in the sequential arm (46%; 95% CI, 30%–61%). For those who had Burkitt-like lymphoma and other highly aggressive subtypes, CR occurred in 10 of 16 patients in the concurrent arm (63%; 95% CI, 35%–85%) and 9 of 11 patients in the sequential arm (82%; 95% CI, 48%–98%). The study was not powered to evaluate differences in patient outcomes between study arms based upon histology (DLBCL versus BL).

At the 2011 ASCO Annual Meeting, Evans et al. [24] reported on their single-institution experience using both R-Hyper-CVAD (*n* = 7) and R-EPOCH (*n* = 14) in HIV-BL. CR was achieved in 43% of patients treated with R-Hyper-CVAD patients, versus 71% in those treated with R-EPOCH. Febrile neutropenia was observed in 86% versus 29% of patients and tumor lysis syndrome (TLS) in 43% versus 14% of patients for those treated with R-Hyper-CVAD and R-EPOCH, respectively. The authors interpreted this data as suggestive of the possibility that R-EPOCH may result in a higher rate of CR and lower rate of infectious complications and TLS, when compared to R-Hyper-CVAD.

### 2.4. PETHEMA Regimen

The Spanish cooperative group Programa Espanol de Tratamiento en Hematologıa (PETHEMA) reported in 2008 their outcomes in HIV-BL patients, as treated identically to BL patients without HIV [25]. Their regimen was based upon one devised and reported by the German Multicenter Study Group for the Treatment of Adult Acute Lymphoblastic Leukemia (GMALL) [26] and relied on a short, intense, non-cross-resistant cocktail of cytotoxic agents, along with rituximab (a total of twelve agents are included in the protocol). In the PETHEMA trial, although three HIV-BL patients died of infectious causes, the remaining 16 (84%) achieved CR. This regimen may involve a higher incidence of toxicity, as 18% of HIV-negative patients and 32% of HIV-BL patients (*P* = NS) were not able to receive the full 6 cycles of planned therapy. Nevertheless, neither the 2-year DFS (93%; 95% CI, 82%–99% in HIV-negative and 87%; 95% CI, 72%–99% in HIV-positive) nor the 2-year OS (82%; 95% CI, 65%–99% in HIV-negative and 73%; 95% CI, 54%–92% in HIV-positive) differed significantly. CNS involvement was rare in this series (3/17 among HIV-negative; 1/19 among HIV-positive), though it was reported by the authors as not impacting outcome.

Expanded results from use of this regimen in 72 HIV-BL patients were reported at the ASH 2010 Annual Meeting [27]. Complete response (CR) was attained in 49 pts (81%), 7 (12%) died in induction, and 4 (7%) were resistant. No relapses were observed after a median follow-up of 2.6 years. In a multivariate analysis, CD4 count <200/mL and involvement of 2 or more extranodal areas predicted for inferior rates of CR, while CD4 count <200/mL and Eastern Cooperative Oncology Group (ECOG) performance status (PS) greater than 2 predicted for inferior OS.

## 3. Areas of Uncertainty

### 3.1. Rituximab

The use of rituximab in patients with ARL has garnered controversy since the reporting of AMC 010 [28], in which the risk of infection-related deaths appeared to offset any benefit of the drug in patients with ARL (80% of which had DLBCL histology) treated with a chemotherapy backbone of cyclophosphamide, doxorubicin, vincristine, and prednisone (CHOP). In AMC 034 [23], among eight patients with baseline CD4 counts below 50/*μ*L, three (38%) experienced treatment-related infectious fatalities. A retrospective review of HIV-BL patients treated with aggressive regimens has questioned whether rituximab improves outcomes [29].

However, in AMC 034, which had a relatively larger proportion of patients with HIV-BL as compared to AMC 010, rates of serious infection, and of infection-related death, were modest and not greater than what would be expected with EPOCH alone. The incorporation of rituximab into the PETHEMA regimen corroborates the idea that high cure rates with modest rates of infectious complications can be achieved in patients with HIV-BL [27]. Similarly, the safety data of rituximab plus EPOCH reported in patients with HIV-DLBCL [20], and implicit in the 100% survival preliminarily reported for those with HIV-BL [22], indicates that rituximab can be safely given to patients with ARL, particularly to those in whom baseline CD4 count is greater than 50/*μ*L. In the HIV-negative population, the addition of rituximab appears to improve outcomes in BL when added to intensive multiagent chemotherapy [30]. Ultimately, in the absence of randomized data in patients with HIV-BL (which is uniformly CD20+), the overwhelming evidence of the safety and efficacy of the addition of rituximab to chemotherapy in both HIV-DLBCL and HIV-BL warrants its ongoing inclusion in protocols for all patients with ARL.

### 3.2. Relapsed/Refractory Disease and Stem Cell Transplant

No prospective data is available regarding the treatment and outcome of relapsed and/or refractory (rel/ref) HIV-BL. The AMC has conducted a retrospective study of patients with ARL treated at member institutions [31] and found that of 12 patients with rel/ref HIV-BL, none received stem cell transplantation (SCT), and only one patient survived beyond one year, only to succumb to malignancy. A French report of 14 patients with ARL treated with autologous SCT (ASCT) included two patients with HIV-BL, both of whom died within a month of transplant due to refractory disease [32]. On the other hand, the City of Hope reported that four of five HIV-BL patients were alive in CR at between 20 and 60 months from time of ASCT [33]. In an ongoing Italian trial of patients with HIV-BL, with a provisional intensification phase that includes ASCT [34], thirteen patients have been treated with 38-day induction consisting of methylprednisolone, cyclophosphamide, vincristine, rituximab, methotrexate, VP-16, and doxorubicin, with IT prophylaxis. Those with <CR after induction were referred for ASCT. They report successful transplantation of three such patients, though specific data regarding EFS and OS in this small population remains unclear. Another small series documenting the feasibility of ASCT in ARL [35] did not specify outcomes for HIV-BL patients. Even in the HIV-negative population, data regarding management is scarce. A retrospective evaluation that included 32 patients with rel/ref BL [36] found that ASCT was associated with 3-year OS of 37% in chemosensitive disease, but only 7% in chemoresistant disease, with most patients in the latter group relapsing and dying within six months.

### 3.3. Older Patients

Patient's age above 40 years has been recognized as a risk factor for poor outcome in BL since at least 1990 [8]. It also bears noting that, while Magrath et al. [13] achieved a 100% 2-year OS with CODOX/M-IVAC when treating adults with median age of 25, a similar regimen, when applied to patients with median age of 47 [15], yielded a seemingly inferior 2-year OS of 71%. Nonetheless, a recent review of HIV-negative BL suggests that, since the year 2000, a higher proportion of patients above 40 are being included in clinical trials and the gap in their prognosis is narrowing [37]. Although this might suggest that these “older” patients ought to be treated similarly to younger adults, it remains unclear whether this holds true for those with HIV-BL. The reports in patients with HIV-BL that support the use of Hyper-CVAD [10], DA-EPOCH-RR [22], and the PETHEMA regimen [25] have each included patients over 40, though none provided outcome data specific to this subset. The median age of patients enrolled in the ongoing AMC 048 trial, as of last report, was age 40 [38], so this may permit further comparison between HIV-BL patients above and below this age. Consideration of patient age is likely to increase in the future, given the recent documented increase in the number of persons over 40 living with HIV, which now account for over 40% of the HIV-positive population in the United States [39].

### 3.4. Central Nervous System Involvement

In each of the HIV-BL-specific series discussed above (Table 1), the incidence of reported CNS involvement was low, and no clear conclusions are apparent with regard to the clinical impact of CNS involvement at the time of diagnosis. One controlled trial that predated modern chemotherapy sought to address the role of CSF prophylaxis in BL (*n* = 10 each in control and treatment arms; all patients HIV-negative). In this trial, there was no difference between the arms in terms of subsequent development of CNS disease [40]. However, given the propensity of BL to relapse in with CNS involvement, the poor outcomes seen in such cases [5, 41], and the relatively low risk of added toxicity observed with the addition of CNS prophylaxis, all recent trials/series have employed aggressive CNS-directed prophylaxis with IT chemotherapy.

### 3.5. Antiretroviral Therapy and Supportive Care

The use of HAART appears to improve response to chemotherapy and clinical outcomes when used in ARL [42] and has been shown as early as 1996 to be safely administered with infusional CDE (cyclophosphamide, doxorubicin, and etoposide) in patients with HIV-related NHL [17]. Although controlled data is lacking as to the appropriate use of HAART in HIV-BL, HAART has been included in patients treated with Hyper-CVAD [10]; the excellent results achieved with the PETHEMA regimen [25], wherein HAART was mandatory, seem to weigh in favor of its use. On the other hand, it bears noting that the NIH has shown that safety and efficacy can be maintained with suspension of HAART in patients with HIV-related NHL, during the administration of aggressive chemotherapy [18]. Use of Zidovudine is avoided in HAART regimens for ARL patients due to the risk of myelosuppression, and deleterious interaction between HAART agents and chemotherapy, such as that between tenofovir and methotrexate, have been documented [43].

 HIV patients are prone to development of BL at higher CD4 counts as compared to other AIDS-associated malignancies [44]. However, lower CD4 counts may have contributed to the higher rates of infectious deaths observed both in HIV-BL patients treated with Hyper-CVAD [10] and in ARL patients treated on AMC-010 with R-CHOP [28]. Subsequent trials [20, 21, 23] seem to clearly support the safety and efficacy of aggressive chemotherapy, including rituximab, in patients with CD4 counts greater than 50/*μ*L. Rituximab should be held in patients with very low CD4 counts (<50/uL) due to the increased risk of infections associated with this drug in this subset of patients. Patients on treatment with rituximab with active hepatitis B (HBV) infections should be treated concurrently with lamivudine to prevent hepatitis B reactivation. However, single-agent HBV prophylaxis in the absence of HAART promotes the emergence of resistant strains of HIV, and the use of HAART should therefore be strongly recommended in this population, in accordance with US Department of Health & Human Services Guidelines [45]. Routine supportive care for HIV-BL includes prophylaxis against tumor lysis, CNS disease, and opportunistic infections, especially pneumocystis carinii, fungal infections and mycobacterium avium-intracellulare. The use of granulocyte colony-stimulating factors is also recommended beginning 24–48 hours after treatment until postnadir recovery of white blood cell counts given the myelosuppressive potential of BL chemotherapy regimens.

## 4. Conclusions

No available randomized data directly compare currently used regimens in BL in either the HIV-positive or HIV-negative populations. Nevertheless, the trials described in this paper suggest that patients with HIV may be treated with similar regimens as their HIV-negative counterparts along with appropriate supportive care. The exception to this are HIV patients with baseline CD4 counts below 50/*μ*L, as these patients have historically been either excluded from receiving rituximab in clinical trials or shown to be at significant risk for treatment-related mortality [23].

It remains important to distinguish HIV-BL from HIV-DLBCL, just as it is essential to distinguish the two histologies in the HIV-negative population. Those with HIV-BL, for instance, have distinct oncogenic mechanisms [4], higher rates of CNS involvement [18], and more aggressive clinical course that warrants more intensive therapy [46], as compared to those with HIV-DLBCL. As a result of these differences, the regimens for HIV-BL tend to carry greater risks of TRM as compared to those used for HIV-DLBCL, but with lower rates of late relapse, resulting in long-term survival rates that tend to approximate 50–80% for either histology [10, 16, 18, 20, 23–25, 34].

 With respect to chemotherapy regimens used in untreated HIV-BL, several conclusions seem reasonable: (1) though the number of patients was very small, Hyper-CVAD appears effective, but may carry an increased risk of mortality in patients not receiving HAART or with poor performance status. The addition of rituximab to Hyper-CVAD has not shown significantly improved patient outcomes in HIV-BL. (2) CODOX-M/IVAC is associated with high CR rates but unacceptable neurotoxicity and myelosuppression. Modification of CODOX-M/IVAC by decreasing the dose of methotrexate, capping the vincristine dose, modifying the cyclophosphamide dose schedule, and increasing doxorubicin doses have reduced toxicity while preserving efficacy. The addition of rituximab to the modified CODOX-M/IVAC regimen is under investigation by the AMC. (3) Although the PETHEMA regimen achieved excellent clinical outcomes, the addition of rituximab to this or a similar regimen has not been studied prospectively. In addition, the mandatory use of HAART in this trial demonstrated the feasibility of such an approach. (4) Results for the infusional DA-EPOCH chemotherapy backbone (as reported by both the NIH and AMC) are promising, with high CR and OS rates, and warrant further prospective investigation in larger trials.

In addition, the following tenets of effective therapy, though lacking the support of randomized data, seem warranted: patient screening for CNS involvement along with appropriate CNS prophylaxis is recommended and is particularly important with DA-EPOCH, as none of the agents in this regimen achieves clinically meaningful CNS penetration. Insufficient data exist to support HDT-ASCT as initial therapy for HIV-BL or as consolidation therapy for those in first CR, though it may benefit a subset of patients with chemosensitive relapsed disease. Supportive care for HIV-BL patients is important and includes HAART, as well as prophylaxis against tumor lysis syndrome, CNS disease, and opportunistic infections. The selection of individual regimens must be made by weighing the risks and benefits for individual patients.

In closing, Table 2 provides in summarized format what we believe are some of the most important principles supported by our review of the existing data for the treatment of HIV-BL.

## Figures and Tables

**Table 1:** Comparison of trials/series including patients with HIV-BL.

Chemotherapy regimen (reference)	Number of patients	Patient characteristics	ORR (%)	CRR (%)	OS/EFS	Comments
Hyper-CVAD [10]	13	6 with BL, 7 with L3-ALL; 31% with preexisting HIV; median CD4: 77/*μ*L 23% CSF+	100	92	48% OS at 2 years	HAART appeared to improve outcome; CNS ppx: alternating MTX and Ara-C × 16 total
CODOX-M/IVAC [14]	8	88% with stage IV disease; median CD4: 149/*μ*L 21% CSF+	N/A	63	57% EFS at 2 years	Impact of HAART unclear; CNS ppx: Ara-C (x2) and MTX with each cycle
DA-EPOCH-R [21]	8	56% with stage III or IV; 76% with extranodal disease; CD4 count data N/A; proportion CSF+ N/A	100	100	96% EFS at 35 mo.	HAART use unclear; CNS ppx: MTX × 6 total
DA-EPOCH-R [23] Concurrent	51*	25% with HIV-BL*; 79% with advanced stage; median CD4: 181–194/*μ*L	88	73	70% OS at 2 years	CRR for HIV-BL: 63% (10/16)
Sequential	55*	CNS ppx and HAART at discretion of treating physician	77	55	67% OS at 2 years	CRR for HIV-BL: 82% (9/11)
PETHEMA [25]	19	58% with stage III or IV; 89% with preexisting HIV; 58% with CD4 count >200/*μ*L 5% CSF+	88	88	77% OS at 2 years	HAART mandatory; CNS ppx: MTX, Ara-C, Dex × 8

HIV: human immunodeficiency virus; HIV-BL: HIV/AIDS-related Burkitt lymphoma; ORR: overall response rate; CRR: complete response rate; OS: overall survival; EFS: event-free survival; L3-ALL: Burkitt cell acute lymphoblastic lymphoma; HAART: highly active antiretroviral therapy; CSF: cerebrospinal fluid; CNS: central nervous system; MTX: methotrexate; Ara-C: cytarabine; Dex: dexamethasone; N/A: not available. Please refer to text for explanation of chemotherapy regimen abbreviations.

*Most patients treated as part of AMC 034 had HIV/AIDS-related DLBCL (see text for further explanation).

**Table 2:** Paradigms in the treatment of HIV/AIDS-related Burkitt lymphoma.

(i) Hyper-CVAD appears effective but may carry an increased risk of mortality in patients not receiving HAART or with poor performance status. The addition of rituximab to Hyper-CVAD has not shown significantly improved patient outcomes in HIV-BL.	
(ii) CODOX-M/IVAC is associated with high CR rates but unacceptable neurotoxicity and myelosuppression. Modification of CODOX-M/IVAC has reduced toxicity while preserving efficacy. The addition of rituximab to the modified CODOX-M/IVAC regimen is under investigation by the AMC.	
(iii) Although the PETHEMA regimen achieved excellent clinical outcomes, the addition of rituximab to this or a similar regimen has not been studied prospectively.	
(iv) Results for the infusional R-EPOCH chemotherapy backbone (as reported by both the NIH and AMC) are promising, with high CR rates, and warrant further prospective investigation in larger trials.	
(v) Patient screening for CNS involvement, along with appropriate CNS prophylaxis, is recommended and is particularly important with the use of R-EPOCH, as none of the agents in this regimen has significant penetration of the CNS.	
(vi) There are insufficient data to support autologous stem cell transplantation as initial therapy for HIV-BL patients, though transplant may benefit a subset of patients with chemosensitive relapsed disease.	
(vii) Supportive care for HIV-BL patients is important and includes HAART as well as prophylaxis against tumor lysis syndrome, CNS relapse, and opportunistic infections.	
